# More Sensitive Identification for Bradykinesia Compared to Tremors in Parkinson’s Disease Based on Parkinson’s KinetiGraph (PKG)

**DOI:** 10.3389/fnagi.2020.594701

**Published:** 2020-11-03

**Authors:** Lina Chen, Guoen Cai, Huidan Weng, Jiao Yu, Yu Yang, Xuanyu Huang, Xiaochun Chen, Qinyong Ye

**Affiliations:** ^1^Department of Neurology, Fujian Institute of Geriatrics, Fujian Medical University Union Hospital, Fuzhou, China; ^2^Institute of Neuroscience, Fujian Key Laboratory of Molecular Neurology, Fujian Medical University, Fuzhou, China

**Keywords:** Parkinson’s disease, the Parkinson’s KinetiGraph, motor symptoms, bradykinesia, fluctuations

## Abstract

The effective management and therapies for Parkinson’s disease (PD) require appropriate clinical evaluation. The Parkinson’s KinetiGraph (PKG) is a wearable sensor system that can monitor the motion characteristics of PD objectively and continuously. This study was aimed to assess the correlations between PKG data and clinical scores of bradykinesia, rigidity, tremor, and fluctuation. It also aims to explore the application value of identifying early motor symptoms. An observational study of 100 PD patients wearing the PKG for ≥ 6 days was performed. It provides a series of data, such as the bradykinesia score (BKS), percent time tremor (PTT), dyskinesia score (DKS), and fluctuation and dyskinesia score (FDS). PKG data and UPDRS scores were analyzed, including UPDRS III total scores, UPDRS III-bradykinesia scores (UPDRS III-B: items 23–26, 31), UPDRS III-rigidity scores (UPDRS III-R: item 22), and scores from the Wearing-off Questionnaire (WOQ-9). This study shows that there was significant correlation between BKS and UPDRS III scores, including UPDRS III total scores, UPDRS III-B, and UPDRS III-R scores (*r* = 0.479–0.588, *p* ≤ 0.001), especially in the early-stage group (*r* = 0.682, *p* < 0.001). Furthermore, we found that BKS in patients with left-sided onset (33.57 ± 5.14, *n* = 37) is more serious than in patients with right-sided onset (29.87 ± 6.86, *n* = 26). Our findings support the feasibility of using the PKG to detect abnormal movements, especially bradykinesia in PD. It is suitable for the early detection, remote monitoring, and timely treatment of PD symptoms.

## Introduction

Parkinson’s disease (PD) is the second most common progressive neurodegenerative disease; it is characterized by motor function and non-motor symptoms (Gronek et al., 2020) and affects approximately 1% of the population aged over 60 years old (Pringsheim et al., 2014). PD is associated with neuronal loss and structural changes in different areas of brain (Beheshti et al., 2020), pathologically characterized by progressive dopaminergic loss in the substantia nigra pars compacta (SNpc) (Zheng et al., 2020). Dopaminergic motor symptoms, including bradykinesia, rigidity, and tremor, are the core features of clinical PD (Grayson, 2016). The motor symptoms of different patients are obviously different, which are divided into different subtypes, such as akineto-rigid type, tremor dominant type, and an equivalent type that had both features (Qian and Huang, 2019). However, bradykinesia is a major motor feature of PD and related to the diagnosis of PD (Postuma et al., 2015). After 5 years of levodopa treatment, 50% of PD patients develop “wearing off” symptoms, motor fluctuations, and dyskinesia (McColl et al., 2002; Stacy et al., 2005; Rosqvist et al., 2018). Sleep disturbance is one of the non-motor symptoms in PD that includes difficulties in falling or staying asleep, sleep fragmentation, and daytime sleepiness with involuntarily daytime naps and RBD (Zhang et al., 2020). The effective management and development of new therapeutic strategies require clinical evaluation, such as PD home diaries and clinical rating scales (e.g., the UPDRS). Although diaries have become important in measuring PD fluctuations, it is difficult to report the changes in clinical states (Stacy et al., 2005; Pahwa et al., 2018). The Unified Parkinson’s Disease Rating Scale (UPDRS) assesses motor performance on a scale of 0 to 4. The higher the score, the higher the severity. The effect of treatment is assessed by a rating scale (such as the UPDRS), but there is no established level corresponding to the treatment goal (Richards et al., 1994; Goetz et al., 2007; Odin et al., 2018). Clinical consultation is a simple phenomenon, yet the symptoms of PD may fluctuate at any time, and the symptoms are not systematically collected and recorded (Ossig et al., 2016b). Therefore, there is a great need to assess the effectiveness of therapeutic interventions in clinical trials and routine clinical care.

Due to the advance of sensor-based and wearable technology in recent decades, motion sensors have spurred the development of the field of objective evaluation in patients with PD (Espay et al., 2016; Godinho et al., 2016; Ossig et al., 2016a; Ramdhani et al., 2018; Ferraris et al., 2019). There are numerous wearable inertial sensors for measuring the motor features of PD (Heldman et al., 2014; Klingelhoefer et al., 2016; Chung et al., 2018; Farzanehfar et al., 2018; Phan et al., 2018; Joshi et al., 2019; Ricci et al., 2020). There is a profoundly important need for biomarkers that reflect the pathogenetic process of PD in order to improve the ability to diagnose and predict (Xu et al., 2019). The digital biomarkers measured by wearable sensors in prodromal PD will be a valuable tool for screening PD in the future (Xie et al., 2019). The Parkinson’s KinetiGraph (PKG) is a wearable sensor system that provides objective ratings of the motor features of PD, including bradykinesia, dyskinesia, tremor, and fluctuations; the PKG also measures sleep-related parameters (Klingelhoefer et al., 2016), and it provides medication dosing reminders for patients. The system has two algorithms that provide movement likelihood scores for dyskinesia or bradykinesia, i.e., the dyskinesia score (DKS) and the bradykinesia score (BKS) (Griffiths et al., 2012). The fluctuating and dyskinesia score (FDS) was developed as a summary score of motor fluctuations and dyskinesias (Tan et al., 2019). It is the logarithm of the sum of the interquartile range of BKS and DKS during the recording period. It has been shown to distinguish clinical fluctuation factors from non-fluctuators (Horne et al., 2015; Odin et al., 2018; Pahwa et al., 2018). When the subharmonics are obvious, PKG can determine the tremor pattern that is significantly larger than the main peak of 3 Hz on the spectrogram. It is helpful to discriminate between tremor and dyskinesia (Braybrook et al., 2016). In a study, the PKG was used to guide treatment as an objective measure, and the results showed that the patient’s clinical scale score was significantly improved, such as UPDRS III and the Montreal Cognitive Assessment (MoCA) (Farzanehfar et al., 2018). The study also demonstrated that the PKG can provide guidance and evaluation for the use of deep brain stimulation (DBS). Moreover, the PKG can also distinguish fluctuations and continuously and objectively monitor the natural course of PD (Horne et al., 2016; Odin et al., 2018; Khodakarami et al., 2019).

The study aimed to assess the correlations between PKG output data and the clinical scores of bradykinesia, rigidity, tremor, and fluctuation. Additionally, the PKG is a valuable tool for objectively and effectively measuring and managing motor symptoms in PD patients.

## Materials and Methods

### Study Cohort

This study was conducted in Fuzhou, China, with approval from the Fujian Medical University Union Hospital Ethics Committee. We have recruited 113 patients in the study, but only 100 patients met the selection criteria and completed assessment. Data were collected from 100 patients with PD according to the UK Parkinson’s Disease Society Brain Bank criteria (Hughes et al., 1992). All patients signed informed consent forms prior to participation. The data collection and evaluation of information were completed by two movement disorder specialists. The patients were divided into two groups according to their H&Y stages (Hoehn-Yahr stage). The patients in H&Y stage 1–2 were in the early-stage group, and patients in H&Y stages 2.5–5 were in the middle-late-stage group.

### Participants

Patients were included if they met the diagnostic criteria for PD and received levodopa treatment. Patients with the following conditions were excluded: (1) severe cognitive dysfunction; (2) other heart, lung, liver, and kidney disease history; (3) non-PD physical disability; and (4) inability to cooperate or complete data collection.

### Initial Examination

All 100 patients were assessed with the following scales: the UPDRS, the Mini-Mental State Examination (MMSE), the MoCA, and the Non-motor Symptoms Scale (NMSS). Seventy-five of 100 patients who have received stable levodopa treatment with a dose of 125mg tid were assessed using the Wearing-off Questionnaire-9 (WOQ-9). Among them, there are 27 patients in the H&Y:1–2 stage and 48 patients in the H&Y:2.5–3 stage. They also provided their history to and underwent an examination by a neurologist (Ye. Q). All patients completed the scales when they thought they were in good condition, i.e., 30 to 60 min after taking the medicine, and the investigators were properly blinded for the scales examination. The clinical scale scores, including UPDRS III total scores, UPDRS III-B scores, UPDRS III-rigidity scores, UPDRS III-T scores, UPDRS II-T scores, and WOQ-9 scores, were analyzed. The items assessing finger taps (item 23), hand movements (item 24), pronation/supination (item 25), leg agility (item 26), and body bradykinesia (item 31) contribute to the UPDRS III-B scores. The items assessing resting tremor (item 20) and postural tremor (item 21) contribute to the UPDRS III-T scores.

### PKG Motor State Measurement

The PKG system includes a data logger (PKG logger) that is worn on the wrist, which was the most severely affected side (Chung et al., 2018). The PKG is approved by the FDA to measure the motor features of PD, including bradykinesia, dyskinesia, tremor, and fluctuations; furthermore, it measures sleep-related parameters and provides reminders for patients to take their medication (Santiago et al., 2019; Pahwa et al., 2020). The PKG provides a series of charts of BKS and DKS collected every 2 min between 6 am and 10 pm over 7 days. Finally, numerical scores for tremor [the Percent Time Tremor (PTT)] and sleep time [Percent Time Immobile (PTI)] were provided in addition to the FDS, BKS, and DKS (Farzanehfar and Horne, 2017).

### Statistical Analyses

Statistical analyses were conducted using Pearson’s correlation test to examine the association between the PKG data and clinical scale scores. The intraclass correlation (ICC) approach was used to examine the test–retest reliability of the PKG and to assess the consistency of the two assessment methods. Independent *t*-tests were used to examine the differences in the characteristics of the early-stage group and middle-late-stage group, and categorical data were compared using χ^2^ tests. Unless mentioned otherwise, all data are displayed as the means ± SD or frequencies (%), and the significance level was set at *p* < 0.05 (two-tailed test).

## Results

### The Clinical and PKG-Related Characteristics of the Participants

Data were obtained from 100 PD patients [40 women (40%)]. The patients were divided into two groups according to the H&Y stage. Among them, 35 patients were early-stage (H&Y: 1–2) patients, and 65 patients were middle-late-stage (H&Y: 2.5–5) patients. However, patients included in the middle-late-stage group were limited to H&Y: 2.5–3. Compared with the early-stage group, the middle-late-stage group had significantly higher mean scores on the UPDRS I, UPDRS II, UPDRS III, and NMSS, and then it is opposite for MMSE and MoCA scores. However, there were no significant differences in the DKS, BKS, PTT scores, PTI scores, or WOQ-9 scores between the two groups. The clinical and PKG-related characteristics of the participants are shown in Table 1.

**TABLE 1:** The clinical and Parkinson’s KinetiGraph (PKG)-related characteristics of the participants.

	All PD	H&Y:1–2	H&Y:2.5–3	*p*
Sample size (M/F)	100 (60/40)	35 (22/13)	65 (38/27)	0.672
Age (years)	66.36 ± 0.92	67.3 ± 8.04	66.46 ± 8.45	0.113
PD duration (years)	5.2 ± 3.41	5.12 ± 2.83	5.25 ± 3.7	0.925
H&Y stage	2 (2–2.5)	2 (1–2)	2.5 (2.5,3)	< 0.001*
UPDRS I	2.61 ± 0.16	2.3 ± 1.33	2.69 ± 1.6	0.002*
UPDRS II	10.72 ± 0.50	8.1 ± 3.63	11.38 ± 3.1	< 0.001*
UPDRS III	31.57 ± 1.3	23.6 ± 10.22	31.38 ± 8.46	< 0.001*
UPDRS III-R	7.34 ± 4.4	5.91 ± 3.86	8.1 ± 4.5	0.017*
UPDRS III-B	14.66 ± 6.5	10.89 ± 6.1	16.69 ± 5.79	< 0.001*
UPDRS III-T	2.86 ± 3.74	2.49 ± 2.05	3.06 ± 4.39	0.465
MMSE	25.87 ± 3.2	27.5 ± 1.84	24.61 ± 3.5	0.001*
MoCA	21.48 ± 3.88	23.5 ± 3.74	19.92 ± 3.33	0.005*
NMSS	39.43 ± 19.56	37 ± 20.12	41.31 ± 19.72	0.009*
WOQ-9^a^	2.77 ± 2.57 (*n* = 75)	2.63 ± 2.51 (*n* = 27)	2.85 ± 2.61 (*n* = 48)	0.659
BKS	32.32 ± 0.704	30.56 ± 6.07	31.92 ± 3.92	0.028*
DKS	1.86 ± 0.30	1.56 ± 1.89	1.17 ± 1.32	0.142
FDS	7.82 ± 0.28	7.7 ± 2.03	7.18 ± 2.11	0.315
PTI (%)	14.98 ± 0.97	14.48 ± 6.38	16.68 ± 7.78	0.917
PTT (%)	6.33 ± 0.88	7.6 ± 10.47	3.98 ± 6.54	0.094

All data are displayed as the means ± SD or frequencies (%). Independent t-tests were used to examine the differences in the characteristics of the early-stage group and middle-late-stage group, and categorical data were compared using χ^2^ tests. *Statistically significant differences (p < 0.05). ^a^Seventy-five of 100 patients were assessed using the WOQ-9 and had received stable levodopa treatment with a dose of 125 mg tid, and there are 27 patients in the H&Y:1–2 stage and 48 patients in the H&Y:2.5–3 stage. M/F, male/female; H&Y, Hoehn-Yahr stage; UPDRS, Unified Parkinson’s Disease Rating Scale; UPDRS I, Part I of UPDRS; UPDRS II, Part II of UPDRS; UPDRS III, Part III of UPDRS; UPDRS III-R, rigidity scores of UPDRS III; UPDRS III-B, bradykinesia scores of UPDRS III; UPDRS III-T, tremor scores of UPDRS III; MMSE, Mini-Mental State Examination; MoCA, Montreal Cognitive Assessment; NMSS, Non-Motor Symptoms Scale. WOQ-9, Wearing off Questionaire-9; BKS, Bradykinesia score; DKS, Dyskinesia score; FDS, Fluctuation and Dyskinesia Score; PTT, the Percent Time Tremor: PTI, Percent Time Immobile.

### The Correlation Between Clinical Scale Scores and PKG-Related Characteristics

**FIGURE 1:**
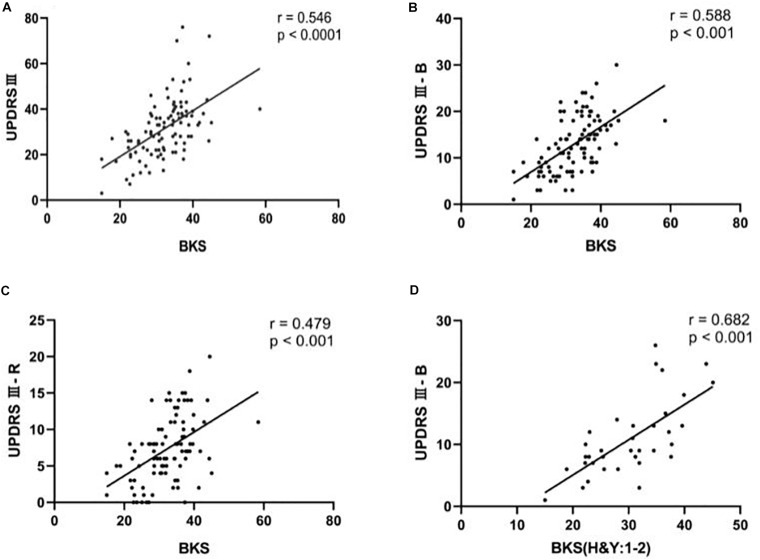
The correlation between clinical scale scores and BKS using Pearson’s correlation test. **(A)** Scatter plots of UPDRS III as a function of BKS for people with Parkinson’s disease (PwP). **(B)** Scatter plots of UPDRS III-B as a function of BKS for the PwP. **(C)** Scatter plots of UPDRS III-R as a function of BKS for PwP. **(D)** Scatter plots of UPDRS III-B as a function of BKS for PwP in the early-stage group (H&Y: 1–2).

In this study, we examined the correlation between the scale scores and the PKG data, and we found that the BKS is moderately correlated with UPDRS III scores, which is shown in Figure 1A (*r* = 0.546, *p* < 0.05), including bradykinesia in Figure 1B (*r* = 0.588, *p* < 0.05) and rigidity in Figure 1C (*r* = 0.479, *p* < 0.05). The Pearson correlation coefficient and the ICC coefficient (ICC = 0.586) were similar for bradykinesia scores and the BKS, but they were slightly different between UPDRS III scores and the BKS (ICC = 0.457). Bradykinesia scores (items 23–26, 31) included finger tap scores (item 23), hand movement scores (item 24), pronation/supination scores (item 25), leg agility scores (item 26), and body bradykinesia scores (items 31). We conducted an analysis of the correlation between these scores and the BKS and found that the correlation coefficients were basically similar (*r* = 0.454–0.557, *p* < 0.05), as shown in Table 2. In addition, an analysis of the correlation between the PTT scores and the tremor scores of the UPDRS was also performed. There were significant correlations between the PTT scores and tremor scores, namely, the UPDRS III-T (*r* = 0.434, *p* < 0.05) and the UPDRS II-T (*r* = 0.269, *p* < 0.05). However, we found that the WOQ-9 had a very weak, non-significant correlation with the DKS and the FDS (*p* > 0.05). The correlation coefficients and *p*-values for these results are summarized in Table 2 and are partially presented in Figures 1A–C.

**TABLE 2:** The correlation between clinical scale scores and BKS by Pearson’s correlation test.

	All patients		H&Y:1–2		H&Y:2.5–3	
	*r*	*p*	*r*	*p*	*r*	*p*
UPDRS III	0.546	<0.001*	0.663	<0.001*	0.445	<0.001*
UPDRS III-R	0.479	<0.001*	0.548	0.001*	0.406	0.001*
UPDRS III-B	0.588	<0.001*	0.682	<0.001*	0.485	<0.001*
Finger taps	0.456	<0.001*	0.603	<0.001*	0.319	0.01*
Hand movements	0.557	<0.001*	0.544	0.001*	0.516	<0.001*
Pronation/supination	0.552	<0.001*	0.623	<0.001*	0.453	<0.001*
Leg agility	0.454	<0.001*	0.618	<0.001*	0.314	<0.001*
Body bradykinesia	0.437	<0.001*	0.534	<0.001*	0.313	<0.001*

*Statistically significant differences (p < 0.05).

### The Correlation Between Clinical Scale Scores and PKG-Related Characteristics (BKS) in Early-Stage Groups

In the correlation analysis between PKG data and individual symptom scores, the bradykinesia scores had the strongest correlation with the BKS. Therefore, we divided the patients into early-stage and middle-late-stage groups and performed correlation analyses. We found that the correlation (*r* = 0.682, *p* < 0.05) between the BKS and bradykinesia scores was significantly higher in the early-stage group than in the middle-late-stage group and the general population, as shown in Figure 1D. The correlation coefficients and *p*-values for these results are summarized in Table 2.

### Comparisons of PKG Data Among People With Parkinson’s Disease (PwP) Across Different Stages

**FIGURE 2:**
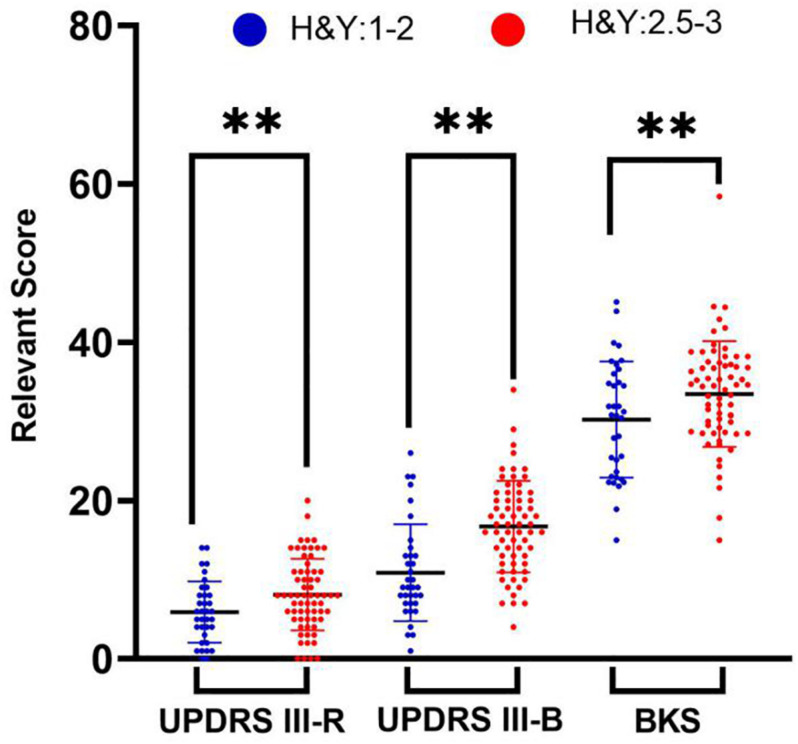
Independent *t*-tests were used to examine the differences in the characteristics of the early-stage group and middle-late-stage group. Comparisons of the rigidity scores, bradykinesia scores, and BKS characteristics of people with Parkinson’s disease in the early-stage group (blue symbols) and middle-late-stage group (red symbols). ^∗∗^Statistically significant differences (*p* < 0.001).

The middle-late-stage group (16.69 ± 5.79) had a significantly higher mean bradykinesia score than the early-stage group (10.89 ± 6.1). Additionally, the early-stage group (30.56 ± 6.07) had a significantly lower mean BKS than the middle-late-stage group (31.92 ± 3.92). There was no statistically significant difference in the scores of tremor items or the PTT between the two groups. These findings reflected the consistency between the scales and the PKG data. The clinical data and PKG data of the participants are shown in Table 1 and Figure 2.

### Percent Time at Levels of Severity (%) Among PwP Across Different Stages

**FIGURE 3:**
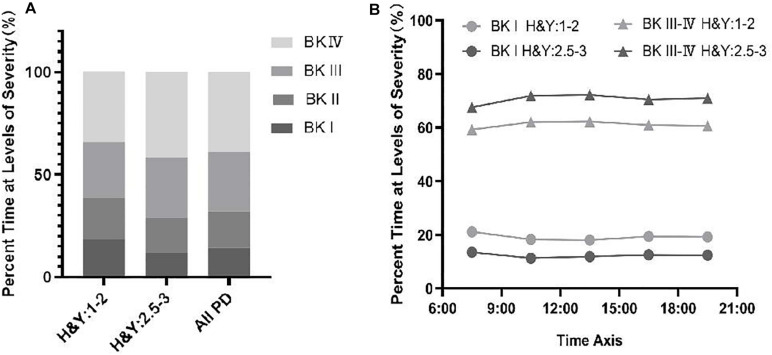
**(A)** Percent time at levels of severity (%) of BKS among PwP across different stages. **(B)** Change in the percentage of time at the severity level (%) in PwP during the day across different stages.

The PKG system divides bradykinesia into four levels, records percent time at levels of severity (%), and can continuously monitor the average value of percent time at levels of severity (%) during the day. In Figure 3A, Parkinson’s patients were dominated by grades BK III–IV, and BK III–IV in advanced patients was higher than in early patients. In Figure 3B, in the two groups of patients, the curve of bradykinesia change is basically the same, but the BK III–IV curve of the late-stage patients is above the early patient group. The results illustrated that bradykinesia was more severe in advanced patients than in early patients.

### Comparisons of PKG Data Among PwP Across Different Side Onset

**FIGURE 4:**
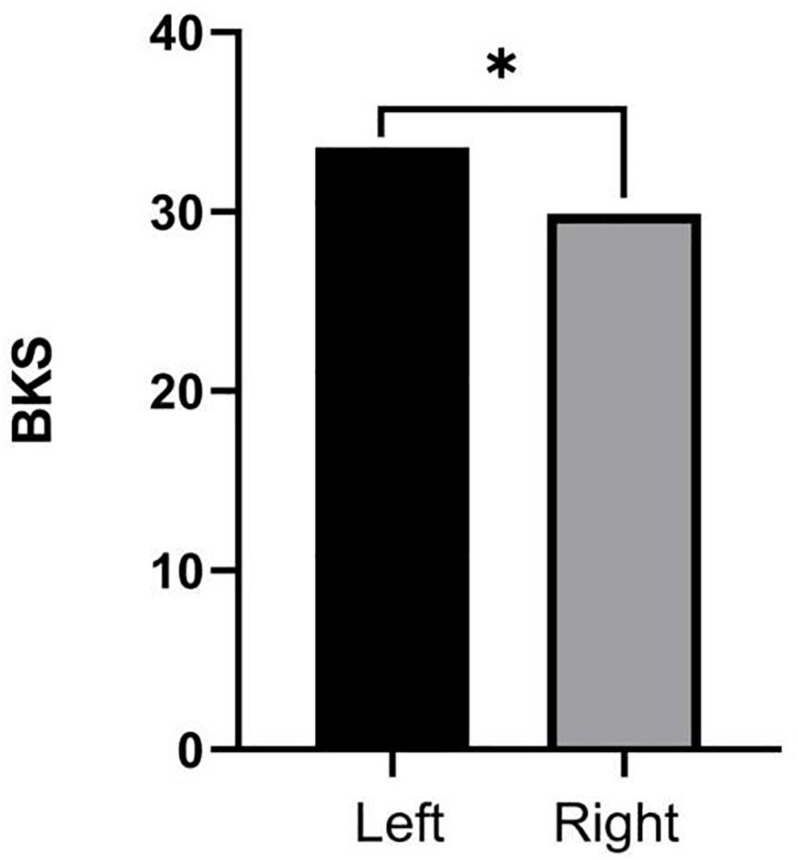
Independent *t*-tests were used to examine the comparisons of BKS among people with Parkinson’s disease (PwP) across different side onset. ^∗^Statistically significant differences (*p* < 0.05).

Our dataset contains information from 65 individuals with PD who have a clear initial onset side (left side of onset: 37; right side of onset: 26). Thirty-four cases of PD on the left-onset side have severe symptoms on left side. Twenty cases of PD on the right-onset side have severe symptoms on right side. We found that BKS in patients with left-sided onset (33.57 ± 5.14, *n* = 37) is more serious than in patients with right-sided onset (29.87 ± 6.86, *n* = 26), which is shown in Figure 4.

## Discussion

Parkinson’s disease mainly manifests as motor symptoms and requires a multidisciplinary treatment approach. Seventy percent of PD patients have unknown or uncontrolled symptoms, and these motor symptoms are treatable. Among the poorly controlled symptoms is bradykinesia, which is often undetected but can be effectively treated. Due to the discrete nature of human visual ability and perception bias, UPDRS can hardly detect subtle changes in disease progression (Ricci et al., 2020). Studies have shown that PKG can accurately identify most of the motor symptoms that can be treated and help make treatment decisions in about 80% of cases where treatment changes (Farzanehfar et al., 2018). In addition, because the data is generated within a few days, the difference in the measurement results of the PKG algorithm is small. In contrast, UPDRS III is only measured once, and a single UPDRS III score cannot capture this fluctuation (Griffiths et al., 2012).

Previous studies have demonstrated that the PKG system could be applied in the routine treatment of PD for therapeutic decision (Horne et al., 2016). Therefore, monitoring motor symptoms with the PKG can be used to assess the dopaminergic transport status of patients (Farzanehfar and Horne, 2017; Pahwa et al., 2018). These results emphasize the potential of PKG to continuously and objectively measure bradykinesia, dyskinesia, tremor, sleep, and motor fluctuations (Griffiths et al., 2012; Odin et al., 2018).

Our study investigates that the correlation between UPDRS III scores and the BKS of 100 patients with PD was significant, although the correlation coefficient (*r* = 0.546) is modest. This is consistent with the results of previously published articles; in addition, we expanded the sample size to 100 patients. Although bradykinesia, tremor, and rigidity each contribute to the UPDRS III score, they may also change independently of each other, leading to overall changes in the UPDRS. Kotschet’s study excluded tremor assessments from the UPDRS III because tremor is not always accompanied by bradykinesia, and its severity may not be related to bradykinesia. The linear correlation produced an *r*^2^ of 0.42 between the median BKS and the revised UDRS III score without tremor assessments in a set of 85 patients (Horne et al., 2016). Based on previous studies, we assessed the scale items separately and then analyzed the relation between BKS and scores of rigidity, tremor, and bradykinesia measured by the UPDRS III. We found that there was a significant correlation between UPDRS III-B scores and the BKS (*r* = 0.588), which is consistent with the correlation between UPDRS III total scores and the BKS. The items assessing finger taps, hand movements, pronation/supination, leg agility, and body bradykinesia contribute to the UPDRS III-B scores. We found that their relevance to the BKS was also meaningful and basically consistent with the above results. These findings also suggested that the correlation between UPDRS III-R scores and the BKS was moderate (*r* = 0.4791). However, the correlation between UPDRS III-T scores and PTT scores was weak (*r* = 0.269). It may be because the scale evaluation is only the evaluation of the patient’s outpatient visit that the patient’s tremor is not captured. The PKG watch monitored continuously to obtain data for 7 days. Therefore, the correlation between UPDRS III-T scores and PTT scores is poor. The findings further illustrate the potential of the PKG to monitor Parkinson’s-related motor symptoms in greater detail than scales, especially bradykinesia. However, we found that the WOQ-9 has a very weak correlation with the DKS and the FDS. This may be because the included patients had fewer motor complications; the average FDS was 7.82 ± 0.28, which is within the FDS reference range (from 7 to 12). Moreover, the WOQ-9 is mainly used to evaluate patients with motor symptoms and non-motor symptoms at the end of the dose. The FDS mainly integrates BKS and DKS data to assess the patient’s movement fluctuations that do not involve the patient’s non-motor symptoms, which may result weak correlations and non-significant results.

In the correlation analysis between PKG data and individual symptom scores, the bradykinesia scores had the strongest correlation with the BKS. Therefore, we divided the patients into early-stage and middle-late-stage groups and performed correlation analyses. We found that the correlation (*r* = 0.682) between the BKS and bradykinesia scores was significantly higher in the early-stage group than in the middle-late-stage group or the general population. Moreover, we compared the BKS, PTT, DKS, and FDS, as measured by the PKG, between the two groups of patients, and we found that the results were consistent with the bradykinesia scores, tremor scores, and motion fluctuation scores derived from the clinical scales. These findings suggest that the PKG has certain advantages in early symptom identification and monitoring of motor symptoms, especially bradykinesia.

Fluctuations are prone to occur in the later stages of PD. The risk factors for sports complications include age of onset, course of disease, severity of disease, treatment time, and dose of levodopa. Prevention of sports complications is important. The PKG watch can continuously monitor Parkinson’s motor symptoms, such as bradykinesia, tremor, and can also monitor movement fluctuations. It can indicate the relationship between medication and symptoms or fluctuations, which is conducive to adjusting drugs, monitoring disease development, optimizing levodopa medication, and controlling the daily dose of levodopa, which may prevent sports complications. The BKS score of the PKG watch can effectively evaluate the symptoms of retardation in PD patients. It has the advantages of simplicity, time, and effort in the follow-up of the changes in the symptoms of retardation in patients and can be promoted in clinical practice. Especially during the COVID-19 epidemic, it can reduce the contact between doctors and patients. However, there are some limitations in our study, such as the range of included patients’ stages being relatively narrow (i.e., stages H&Y:1–3) and the number of early-stage patients being small. Therefore, subsequent studies will require more sample data for further validation.

## Conclusion

Our study indicates that the BKS is significantly correlated with the UPDRS III scores; the correlation is especially strong for bradykinesia and in early-stage patients. Our findings support the feasibility of using a PKG to detect abnormal movements in patients with PD, and it is also suitable for the early identification.

## Data Availability Statement

The raw data supporting the conclusions of this article will be made available by the authors, without undue reservation.

## Ethics Statement

The studies involving human participants were reviewed and approved by Fujian Medical University Union Hospital Ethics Committee. The patients/participants provided their written informed consent to participate in this study. Written informed consent was obtained from the individual(s) for the publication of any potentially identifiable images or data included in this article.

## Author Contributions

QY and XC contributed to conception and design of the study. LC undertook study design, execution of statistical analysis, execution of experimental work, and the review and critique of the manuscript. GC and HW help to organize the research project of the manuscript. JY, YY, and XH review and critiqued the manuscript. All authors contributed to manuscript revision, and read and approved the submitted version.

## Conflict of Interest

The authors declare that the research was conducted in the absence of any commercial or financial relationships that could be construed as a potential conflict of interest.
